# Career choice regret during COVID-19 among healthcare students and professionals in mainland China: a cross-sectional study

**DOI:** 10.1186/s12909-021-02972-6

**Published:** 2021-10-18

**Authors:** Guoyi Yang, Ling Wang, Jia Wang, Zixian Geng, Huixin Liu, Tao Xu

**Affiliations:** 1 Urology Department, Peking University People’s Hospital, No. 11 Xizhimen South Street Xicheng District, Beijing, 100044 China; 2 Nursing Department, Peking University People’s Hospital, No. 11 Xizhimen South Street Xicheng District, Beijing, 100044 China; 3 Department of Clinical Epidemiology and Biostatistics, Peking University People’s Hospital, Beijing, China

**Keywords:** COVID-19;career choice regret, Healthcare professionals;medical students, Psychological resilience, Professional value

## Abstract

**Background:**

The COVID-19 epidemic affected the career choice of healthcare professionals and students. Career choice regret of healthcare professionals and students during COVID-19 outbreak and its affected factors are largely unexplored.

**Methods:**

Convenience sample of nurses, doctors, and medical students were recruited from hospitals and universities nationwide. The data collected including demographic information, professional value before and after the COVID-19 outbreak, the Connor-Davidson Resilience Scale, and career choice regret level by an online questionnaire. Multinominal logistic regression was employed to explore the factors associated with career choice regret.

**Results:**

In total, 9322 participants of convenience sampling were enrolled in, including 5786 nurses, 1664 doctors, and 1872 medical students. 6.7% participants had career choice regret. Multinominal logistic regression analysis showed, compared to participants with no regret, that as levels of psychological resilience increased, the odds of experiencing career choice regret decreased (OR = 0.95, 95% CI 0.94–0.96), while participants with lower professional value evaluation after the COVID-19 outbreak had higher probability to experience career choice regret (OR = 1.55,95% CI 1.50–1.61). Medical students were more likely to regret than nurses (OR = 1.65,95% CI 1.20–2.28), participants whose career/major choice was not their personal ideal had higher risk of experience career choice regret (OR = 1.59,95% CI 1.29–1.96), while participants who were very afraid of the coronavirus had higher risk to experience career choice regret then participants with no fear at all (OR = 2.00,95% CI 1.24–3.21).

As for the medical students, results indicated that medical students major in nursing and undergraduates had higher risk to experience career choice regret compared to medical students major in clinical medicine and postgraduate (Master or PhD), with an odds ratios of 2.65(95% CI 1.56–4.49) and 6.85 (95% CI 2.48–18.91)respectively.

**Conclusions:**

A minority of healthcare professionals and medical students regretted their career choices during the COVID-19 outbreak. Enhance personal psychological resilience and professional value would helpful to reduce career choice regret among healthcare professionals and students during pandemic.

## Background

The coronavirus disease 2019(COVID-19) is a severe acute respiratory infection caused by SARS-CoV-2 which is high transmission. it has spread to 200 countries and has been declared a global pandemic by the World Health Organization (WHO). Globally, as of 24 November 2020, there have been more than 58 million confirmed cases of COVID-19, including 1385 thousand deaths, reported to WHO (1). With the rapid and extensive spread, doctors and nurses have been confronted with mounting challenges that they have not been faced before.

They encounter work difficulties due to lack of resources and threats to the safety of their loved ones (2) and the highest risk of being infected (3). Moreover, the COVID-19 epidemic had negative impacts on the psychology of healthcare professionals. Studies have showed during the COVID-19 outbreak, healthcare professionals dealing with COVID-19 were under increased levels of anxiety, depression, and stress (4, 5). The epidemic also has increased medical students’ perceptual awareness of the high-risk characteristics of medical and health services (6).

Previous studies showed that depressive symptoms and higher levels of burnout were related to decrease medical career interest, increased career choice regret (7–9). CNN News reported that affected by the epidemic, some American nurses were overwhelmed to strike (10).

Although previous studies have focused on career-choice regret among healthcare professionals and medical students, most have not been in the context of the COVID-19 outbreak (8, 11–13). Only one study investigated the impact of COVID-19 outbreak on the career preferences of medical students in China (7), focusing only on a particular specialty but not on a broader range of doctors,nurses and medical students who are potential doctors or nurses. We conducted the following study with the aims of bridging this gap, to depict the situation of career choice regret among healthcare professionals and students during COVID-19 pandemic and to explore strategies to prevent career choice regret among healthcare professionals and students.

## Methods

### Study design, setting, and participants

A cross-sectional study was conducted from April 23 to May 20,2020. Convenient sampling of doctors and nurses in the hospital and medical college students majoring in clinical medicine and nursing in mainland China were recruited nationwide online. All participants were invited to complete the questionnaire online via Questionnaire Star (https://www.wjx.cn). This study complies with the statement of strengthening the reporting of observational studies in epidemiology (STROBE).

### Measurements

A research group with one physician, two nurses and one epidemiologist were set up to develop the questionnaire. The questions and answer options for the preliminary questionnaire were developed according to the research objective and extensive literature review results. Then an expert panel of two clinical experts, two nursing experts and one epidemiologist were invited to review and revise the preliminary questionnaire. Finally, a pilot study among 40 doctors and nurses and 20 students was conducted before the survey. The questionnaire was further modified according to the feedback of the pilot study to make sure all the questions were clear and unambiguous.

The online questionnaire collected the following information, general information, professional value before and after the outbreak of COVID-19, the Connor-Davidson Resilience scale and one question about career choice regret affected by the COVID-19.Details of each part are as described below.

#### General information

The following information were collected from all the participants, including gender, age, educational level, the reason of career choice for healthcare professionals/ major choice for medical students, whether have experienced verbal violence or physical violence during medical practicing, the willingness to participate in treatment or nursing during public health emergencies and the degree of fear of the coronavirus. In addition to the above, doctors and nurses were also asked if they participated in the treatment or nursing of patients with COVID-19, and medical students were asked whether their current major was the first choice, whether have had started internship in the hospital.

#### Professional value before and after the COVID-19 outbreak

This part contains 5 items to investigate the professional value of the participants before and after the outbreak of COVID-19. Each item using a 5- Likert response scale, response options ranged from “non-conformity” to “full conformity” (score brange,5–25) and a higher score indicated lower professional value evaluation.

#### The Connor-Davidson resilience scale (CD-RISC)

The scale was developed by Connor and Davidson (14) and was revised by Yu (15), comprises 3 dimensions (competency, toughness, and adaptability), 25 items rated on a 5-point scale (0–4), with higher scores reflecting greater resilience. The internal consistency coefficient of the questionnaire is 0.89, which has good reliability and validity.

#### Career choice regret

This part contains one question for all the participants “after the outbreak of COVID-19, I regret the choice I made about my career”. Response options were “strongly agree”, “agree”, “neutral”, “disagree” and“strongly disagree”; responses of “strongly agree” or “agree” indicated with career choice regret, “disagree” and“strongly disagree” indicated without career choice regret.

### Statistical analysis

Categorical data are presented as frequencies and percent, continuous data are described by mean and standard deviation or median and interquartile range (IQR) as appropriate. Mann-Whitney U or Kruskal-Wallis H test were performed to test the association of career choice regret with the following categorical variables, identity, gender, whether experienced physical violence during practicing, whether experienced verbal violence during practicing, the reason of career/major choice, major, work intention after graduation, whether current major was the first choice, whether have had started internship in the hospital, whether participated in the treatment or nursing of patients with COVID-19. Test for Linear Trend was used to test the association of career choice regret with the fear level after the COVID-19 outbreak. Paired t test or Wilcoxon signed-rank test were used to measure the change of professional value during COVID-19. Multinomial logistic regression analysis was performed to identify factors associated with regret of choice of career among all the participants, a subgroup analysis was also done among potential healthcare professionals (medical students). All statistical analyses were conducted in SPSS version 26.0 (IBM, Chicago, IL, USA), and *P* < 0.05 was considered to be statistically significant.

### Ethics statement

This study was approved by Peking University People’s Hospital Ethical Committee [No:2020PHB181–01]. The online survey was anonymous. Informed consent was obtained from all subjects or, if subjects are under 18, from a parent and/or legal guardian when they accessed the online survey.

## Results

### Participants’ characteristics

A total of 9322 participants were finally included, consisted of 5786 nurses, 1664 doctors, and 1872 medical students. Among the medical students, 1100 are major in clinical medicine and 772 are major in nursing. Among the healthcare professionals, 2482 were involved in the treatment or nursing the patient with COVID-19, accounting for 33.3%. The average age of participants was 31.65 years; 1796 were male (19.3%) and 7526 were female (80.7%, Table 1).

**Table 1:** Personal and professional characteristics of participants (*n* = 9322)

	healthcare professionals		medical students	
Characteristics	nurses	doctors	Clinical Medicine	nursing
Gender				
Male	192(3.3%)	1018(61.2%)	468(42.5%)	118(15.3%)
Female	5594(96.7%)	646(38.8%)	632(57.5%)	654(84.7%)
Age	33.02 ± 7.42	38.65 ± 8.27	21.60 ± 3.02	20.60 ± 2.81
Educational level				
Below Bachelor degree	1907(33.0%)	60(3.6%)	0	135(17.5%)
Bachelor degree	3765(65.1%)	773(46.5%)	897(81.5%)	598(77.5%)
Master’s degree	114(2.0%)	506(30.4%)	149(13.5%)	37(4.8%)
Doctorate	0	325(19.5%)	54(4.9%)	2(0.3%)
Work intention after graduation				
Tertiary hospital	N/A	N/A	972(88.4%)	642(83.2%)
Primary hospital	N/A	N/A	69(6.3%)	43(5.6%)
Others	N/A	N/A	59(5.4%)	87(11.3%)
Whether current major is the first choice				
Yes	N/A	N/A	1025(93.2%)	456(59.1%)
No	N/A	N/A	75(6.8%)	316(40.9%)
Whether have had started internship in the hospital				
Yes	N/A	N/A	318(28.9%)	431(55.8%)
No	N/A	N/A	782(71.7%)	341(44.2%)
Reason of major/career choice				
Personal ideal				
Yes	996(59.9%)	2048(35.4%)	825(75.0%)	396(51.3%)
No	668(40.1%)	3738(64.6%)	275(25.0%)	376(48.7%)
Family wishes				
Yes	826(49.6%)	2824(48.8%)	567(51.5%)	358(46.4%)
No	838(50.4%)	2962(51.2%)	553(48.5%)	414(53.6%)

### The level of career choice regret among healthcare professionals and potential healthcare professionals

There were 5506 of 9322 respondents (59.1%) reported that they had no regret of their choice of career, while 624 of 9322 respondents (6.7%) reported that they had career choice regret. For the question about whether the outbreak of COVID-19 made them regret the choice they made about their career, 4.5% reported“agree” and 2.5% reported “strongly agree” among healthcare professionals, while for medical students, 3.9% and 1.4% reported “agree” and “strongly agree”.

### Factors associated with career choice regret after the COVID-19 outbreak in a univariate analysis

The healthcare professionals who participated in the treatment or nursing of patients with COVID-19 have lower career choice regret after the COVID-19 outbreak (Z = -2.726, *P* = 0.006). For all the participants, in the univariate analysis (Table 2), career choice regret after the COVID-19 outbreak was associated with identity, gender, having experienced physical or verbal violence, the reason of career/major choice was personal ideal or family will.

**Table 2:** Univariate analysis of influencing factors of career choice regret after COVID-19 outbreak (*n* = 9322)

Variable	I regret the career choice I made					
	strongly disagree	disagree	neutral	agree	strongly agree	P*
Identity						<0.001
Doctor	277	720	542	79	46	
Nurse	1075	2240	2071	259	141	
Student	391	803	579	73	26	
Gender						0.027
Male	353	764	543	92	44	
Female	1390	2999	2649	319	169	
Whether experienced physical violence						<0.001
Yes	282	622	592	125	72	
No	1461	3141	2600	286	141	
Whether experienced verbal violence						<0.001
Yes	910	2174	1945	301	160	
No	833	1589	1247	110	53	
The reason of career/major choice is personal ideal						<0.001
Yes	1122	1954	1007	110	72	
No	621	1809	2185	301	141	
The reason of career/major choice is family will						<0.001
Yes	769	1880	1607	213	106	
No						
Afraid of coronavirus degree						<0.001
Very afraid	78	77	118	35	36	
Afraid	175	420	488	76	33	
General afraid	607	1842	1687	194	85	
Not afraid	518	1125	708	82	34	
Not afraid at all	365	299	191	24	25	

Note:*P for trend

As for medical students, univariate analysis results showed that, career choice regret after the COVID-19 outbreak was associated with their current major, work intention after graduation, whether their current major was the first choice, whether have had started internship in the hospital, willingness to participate in treatment or nursing facing public health emergencies (Table 3).

**Table 3:** Factors of associated career choice regret after COVID-19 outbreak among medical students(*n* = 1872)

Variable	I regret the career choice I made					
	strongly disagree	disagree	neutral	agree	strongly agree	P*
Educational level						<0.001
Below Bachelor degree	39	47	43	4	2	
Bachelor degree	326	649	457	44	19	
Above Bachelor degree	26	107	79	25	5	
Major						<0.001
Nursing	101	306	310	39	16	
Clinical medicine	101	306	310	39	16	
Work intention after graduation						<0.001
Tertiary hospital	349	716	482	48	19	
Primary hospital	24	47	32	8	1	
Others	18	40	65	17	6	
Whether major is the first choice						<0.001
Yes	360	655	397	52	17	
No	31	148	182	21	9	
Whether is interning in the hospital						<0.001
Yes	125	323	243	45	13	
No	266	480	336	28	13	
For public health emergencies, will you participate in the treatment or nursing?						<0.001
Definitely will	216	206	97	12	10	
Will	135	397	228	27	4	
Not necessarily	37	197	238	32	6	
Not will	1	3	14	2	4	
Definitely not	2	0	2	0	2	

### Factors associated with career choice regret after the COVID-19 outbreak in a multi-factor analysis

Multinominal logistic regression results (Table 4) showed that as levels of psychological resilience increased, the odds of experiencing career choice regret decreased (OR = 0.95,95% CI 0.94–0.96), while participants with higher professional value score (means lower professional value evaluation) had higher probability to experience career choice regret (OR = 1.55,95% CI 1.50–1.61). Medical students were more likely to regret about their career choice than nurses (OR = 1.65,95% CI 1.20–2.28), participants whose career/major choice was not according to their personal ideal had higher risk of experience career choice regret (OR = 1.59,95% CI 1.29–1.96), participants who have not experienced physical violent medical incidents had lower probability to experience career choice regret (OR = 0.67, 95% CI 0.54–0.85), while participants who were very afraid of the coronavirus had higher risk to experience career choice regret then participants with no fear at all (OR = 2.00, 95% CI 1.24–3.21).

**Table 4:** Factors associated with career choice regret after the COVID-19 epidemic in multinominal logistic regression(n = 9322)

Career choice regret		β	SE	Wald	OR(95%CI)	*P*
With career choice regret	Intercept	−5.58	0.55	103.16		<0.001
	Total score of psychological resilience					
		−0.05	0.00	131.32	0.95(0.94–0.96)	<0.001
	Age	−0.01	0.01	2.12	0.99(0.98–1.00)	0.146
	Total score of professional value after the COVID-19 outbreak					
		0.44	0.017	652.894	1.55(1.50–1.61)	<0.001
	Change of professional value					
		−0.15	0.02	48.63	0.86(0.82–0.90)	<0.001
	Identity					
	Student	0.50	0.16	9.48	1.65(1.20–2.28)	0.002
	Doctor	−0.30	0.16	3.47	0.74(0.54–1.02)	0.063
	Nurse	reference				
	Gender					
	Female	−0.17	0.15	1.38	0.84(0.63–1.12)	0.24
	Male	reference				
	The reason of career/major choice is personal ideal					
	No	0.47	0.11	18.99	1.59(1.29–1.96)	<0.001
	Yes	reference				
	The reason of career/major choice is family will					
	No	0.04	0.09	0.20	1.04(0.87–1.25)	0.652
	Yes	reference				
	Whether experienced physical violence					
	No	−0.40	0.12	11.45	0.67(0.54–0.85)	0.001
	Yes	reference				
	Whether experienced verbal violence					
	No	0.12	0.12	1.00	1.13(0.89–1.43)	0.317
	Yes	reference				
	Afraid of coronavirus degree					
	Very afraid	0.69	0.24	8.20	2.00(1.24–3.21)	0.004
	Afraid	0.39	0.20	3.64	1.47(0.99–2.18)	0.056
	General afraid	0.12	0.18	0.42	1.12(0.79–1.59)	0.515
	Not afraid	−0.13	0.19	0.43	0.88(0.60–1.29)	0.514
	Not afraid at all	reference				
neutral	Intercept	−1.87	0.30	39.15		<0.001
	Total score of psychological resilience					
		−0.04	0.00	294.02	0.96(0.95–0.96)	<0.001
	Age	−0.02	0.00	18.18	0.98(0.98–0.99)	<0.001
	Total score of professional value after the COVID-19 outbreak					
		0.25	0.01	687.12	1.29(1.26–1.31)	<0.001
	Change of professional value					
		−0.04	0.01	12.35	0.96(0.93–0.98)	<0.001
	Identity					
	Student	0.16	0.08	3.50	1.17(0.99–1.38)	0.061
	Doctor	0.02	0.09	0.03	1.02(0.86–1.20)	0.855
	Nurse	reference				
	Gender					
	Female	0.08	0.08	1.12	1.09(0.93–1.27)	0.29
	Male	reference				
	The reason of career/major choice is personal ideal					
	No	0.60	0.06	120.18	1.82(1.64–2.03)	<0.001
	Yes	reference				
	The reason of career/major choice is family will					
	No	0.07	0.05	1.62	1.07(0.97–1.18)	0.203
	Yes	reference				
	Whether experienced physical violence					
	No	−0.01	0.07	0.01	0.99(0.86–1.15)	0.934
	Yes	reference				
	Whether experienced verbal violence					
	No	0.25	0.06	17.09	1.28(1.14–1.44)	<0.001
	Yes	reference				
	Afraid of coronavirus degree					
	Very afraid	0.18	0.17	1.09	1.19(0.86–1.65)	0.296
	Afraid	0.55	0.12	22.24	1.73(1.38–2.17)	<0.001
	General afraid	0.49	0.10	24.27	1.63(1.34–1.98)	<0.001
	Not afraid	0.22	0.11	4.50	1.25(1.02–1.54)	0.034
	Not afraid at all	reference				

# The reference category is without career choice regret

As for the medical students, multinominal logistic regression analysis results (Table 5) indicated that medical students major in nursing and undergraduates had higher risk to experience career choice regret compared to medical students major in clinical medicine and postgraduate (Master or PhD), with an odds ratios of 2.65(95% CI 1.56–4.49) and 6.85(95% CI 2.48–18.91) respectively.

**Table 5:** Factors associated with career choice regret after the COVID-19 epidemic in multinominal logistic regression among medical students (*n* = 1872)

Career choice regret		B	SE	Wald	OR(95% CI)	*P*
With career choice regret	Intercept	−2.37	0.57	17.12		<0.001
	Major					
	Nursing	0.97	0.27	13.03	2.65(1.56–4.49)	<0.001
	Clinical Medicine	reference				
	Work intention after graduation					
	Tertiary hospital	−1.52	0.29	27.16	0.22(0.12–0.39)	<0.001
	Primary hospital	−0.62	0.45	1.90	0.54(0.22–1.30)	0.168
	Others	reference				
	Whether is interning in the hospital					
	No	−0.2110	0.27	0.62	0.81(0.48–1.37)	0.433
	Yes	reference				
	Whether major is the first choice					
	No	0.47	0.27	3.04	1.59(0.94–2.69)	0.081
	Yes	reference				
	Educational level					
	Below Bachelor degree	0.48	0.49	0.96	1.61(0.62–4.16)	0.327
	Bachelor degree	1.92	0.52	13.80	6.85(2.48–18.91)	<0.001
	Master’s degree or doctorate	reference				
neutral	Intercept	−0.83	0.30	7.73		0.005
	Major					
	Nursing	0.72	0.13	29.62	2.05(1.58–2.65)	<0.001
	Clinical Medicine	reference				
	Work intention after graduation					
	Tertiary hospital	−0.72	0.20	13.43	0.49(0.33–0.71)	<0.001
	Primary hospital	−0.69	0.29	5.66	0.50(0.29–0.89)	0.017
	Others	reference				
	Whether is interning in the hospital					
	No	0.17	0.14	1.46	1.18(0.90–1.54)	0.226
	Yes	reference				
	Whether major is the first choice					
	No	0.62	0.14	20.63	1.86(1.42–2.44)	<0.001
	Yes	reference				
	Educational level					
	Below Bachelor degree	0.15	0.23	0.42	1.16(0.74–1.80)	0.518
	Bachelor degree	0.76	0.26	8.24	2.13(1.27–3.56)	0.004
	Master’s degree or doctorate	reference				

# The reference category is without career choice regret

## Discussion

This nationwide research investigated the current situation and influencing factors of the career choice regret of healthcare professionals and medical students during the COVID-19 epidemic. Although the epidemic has brought challenges to healthcare, but our research showed only 6.7% healthcare professionals and medical students regret about their career choice. This situation is similarly to previous pandemic (16). Despite the initial shock, the health professionals in China appear to exhibit high levels of commitment and professionalism. The increasing knowledge about preventing and dealing with the disease, and the development of more specific procedural and treatment protocols, alongside educational activities, contributed to improving the morale of healthcare workers dealing with the pandemic.

Our research shows that the degree of regret of medical students’ career choice is lower than that of medical staff, and it is statistically significant. Among medical students, those who have had started internship have more risk to experience career choice regret than those who have not entered clinical internship. It is possible that the COVID-19 pandemic resulted in cancellation of medical student clinical rotations (17). Therefore, educators should intervene and cultivate their professional values after students started internship, and targeted efforts by medical schools to address these concerns through enhanced virtual curriculum development and advising strategies will become increasingly important. So as to reduce their professional regret and reserve talents for the medical career.

The multivariate analysis showed that the professional value was the influence factor of career choice regret during the COVID-19 outbreak. The population with higher professional value showed lower level of regret. Although the professional value has declined slightly after the epidemic, it was still at a high level, and the impact on career choice regret was still positive. Professional value identification plays an important role in career choice. Opportunity to help and care for others as the core professional value of the medicine was the main reason of the students and nurses to undertake nursing (18, 19), and the realization of the professional value of healthcare professionals enables them to insist on their own choice (20) and even to recommend their relatives to work in the health center (21). Therefore, cultivating the professional value of medical staff in peacetime was more conducive to reducing the impact on the career choice regret of medical staff after major public health incidents.

When the COVID-19 outbreak, healthcare professionals directly participated in the treatment or nursing of the patient with COVID-19. They were facing great mental and physical pressure. Regarding the psychological suffering of individuals, an important key psycho-social factor was psychological resilience. Our research showed that the stronger a person’s psychological resilience was, the lower his regret for career choices when facing the COVID-19 epidemic. Resilience plays a decisive role in the response of individuals under pressure and can help them deal with difficulties and adverse circumstances more effectively (22). The previous study showed psychosocial strengths play a significant role in subsiding the risk associated with severity of disease when facing COVID-19 (23). Therefore, it is believed that higher levels of resilience can protect a person from pressure and reduce the risk of regretting career choices.

## Conclusion

During COVID-19 outbreak in China, the degree of career choices among healthcare professionals and medical students was low. Career intention is personal ideal, experienced physical or verbal violence, higher psychological resilience, higher professional value after the epidemic, lower change of professional value were associated with a lower career choice regret. Medical students’ career choice regret was lower than the health professionals. For the medical students, having entered the clinical practice stage, major was not the first choice for college entrance examination volunteers, work intention after graduation wasn’t in hospital had higher level of regret.

### Limitations of this study

Our study is limited in several ways. First, as a cross-sectional design, this study could only evaluate the career choice regret at the time without the longitudinal observation of the subjects, so follow up is warranted in the future. Second, the survey used convenience sample recruited online, which could result in selection bias. However, the findings of this study may have some generalizability given the large sample and with the 50 EPV(50 events per predictor variable) of the multinomial logistic regression, we believe the regression model have good predictive performance. Third, The subjects included in this study were those who are currently doctors, nurses, or medical students after the COVID-19 outbreak, healthcare professionals who have changed careers after the COVID-19 outbreak were not included in this survey, also we did not collect the information about the participants’ former career, there might be some participants who were not healthcare professionals before and engage in healthcare after the COVID-19 outbreak, which might causes bias of the results. Fourth, although we did a literature review and discussed with experts at the proposal stage, there could be some potential unobserved confounding factors that were not controlled.

## Data Availability

The datasets used and/or analysed during the current study are available from the corresponding author on reasonable request.
